# What GPs do to meet accreditation standards – implementation activities and perceived improvements attributed to general practice accreditation

**DOI:** 10.1186/s12875-022-01864-y

**Published:** 2022-10-15

**Authors:** Cecilie Mølgaard, Flemming Bro, Anna Mygind

**Affiliations:** 1 Department of Public Health, Aarhus University, Bartholins Allé 2, DK-8000 Aarhus C, Denmark; 2 Research Unit for General Practice, Bartholins Allé 2, DK-8000 Aarhus C, Denmark; 3 Grangårdsvej 58, 9530 Støvring, Denmark

**Keywords:** General practitioners, Primary care, Accreditation, Implementation science, Normalization process theory, Quality improvement, Prescriptions, Emergency treatment, Cross-sectional studies, Denmark.

## Abstract

**Background:**

Healthcare accreditation is a widely implemented tool used to enhance the quality of care and underpin quality control. However, research is sparse on the accreditation process in general practice. The aim of this study was to explore how team-based implementation activities preceding accreditation were associated with self-perceived improvements in emergency preparedness (preparedness for urgent disease and cardiac arrest) and handling of prescription renewals in Danish general practice.

**Methods:**

GPs (general practitioners) completed a questionnaire exploring practice-team activities conducted to implement two specific accreditation standards and the related improvements as perceived by the GPs. The following implementation activities were selected, inspired by Normalization Process Theory: *Common understanding* (obtaining a common understanding of the purpose of implementing changes according to the accreditation standard), *key person* (assigning a key person responsible for working with the standard), and *easy integration* (finding it easy to integrate changes into existing working procedures). Data were analysed with logistic regression, and adjusted analyses included practice type, number of GP partners, number of staff, training site for junior GPs and administrative region.

**Results:**

The total response rate was 74% (n = 920). Around 80% of the clinics reported having conducted team-based implementation activities. Almost half of the clinics (48%) reported perceived improvements in the emergency preparedness, and 30% reported perceived improvements in the handling of prescription renewals. Obtaining a *common understanding* was found to have a strong, significant association with perceived improvements in the emergency preparedness (OR = 5.07 (3.06–8.40)) and handling of prescription renewals (OR = 3.66 (2.07–6.46)). *Easy integration* of changes was also significantly associated with improvements in both emergency preparedness (OR = 1.88 (1.24–2.85)) and handling of prescription renewals (OR = 2.34 (1.44–3.79)), whereas assigning a *key person* was only significantly associated with improved emergency preparedness (OR = 1.95 (1.19–3.19)).

**Conclusion:**

Clinical quality initiatives that involve collaboration within a practice team are more likely to cause improvements if specific team-based implementation activities are conducted. It is particularly important to facilitate a common understanding of the purpose of the initiative. Therefore, external support for quality initiatives aiming at the practice level in general practice should facilitate such team-based activities.

**Supplementary Information:**

The online version contains supplementary material available at 10.1186/s12875-022-01864-y.

## Background

Accreditation is defined as an evaluation of an organisation by an accreditation agency based on a set of predefined accreditation standards [1]. The purpose of accreditation is to improve the quality and safety of care and to underpin quality control [2, 3]. Accreditation was introduced in the primary healthcare sector in Australia in the 1990s and has been used in the secondary healthcare sector since the beginning of the 20th century [4]. Since then, a number of countries, including the USA, New Zealand and many European countries, have developed accreditation programmes for general practice [2]. Despite the widespread use of healthcare accreditation, research on its implementation and influence on clinical practice in general practice is sparse [2].

In Denmark, a mandatory accreditation programme for general practice was rolled out in 2016–2018 [5]. The Danish Institute for Quality and Accreditation in Healthcare (IKAS), which is an impartial institution, developed and managed the accreditation programme. The standards and indicators were developed by IKAS in collaboration with representatives from the Organization of General Practitioners in Denmark, the Danish College of General Practitioners, Danish Regions, Danish Patients and the Danish Association of Practicing Medical Specialists [6]. The clinics were advised one year in advance about the date of their accreditation survey and were offered support through training courses and workshops, and they were offered on-line information and access to consultants [7]. On this date, the clinic received a visit from two surveyors: a general practitioner (GP) and a practice staff. After the visit, the surveyors prepared a report based on their assessment; this report was used by the accreditation board to decide whether the clinic was eligible for accreditation [5]. The clinics were evaluated based on 16 quality standards containing indicators about professional quality, organisational quality and patient-perceived quality [5]. The standards formed a framework intended to stimulate reflection and inspire quality improvement activities [8]. However, another purpose of the programme was quality control, and many GPs were sceptical and perceived the mandatory accreditation programme as an external control tool [9]. Each clinic received 20,000 Danish kroner (approximately €2650) per GP in the clinic for their participation in the accreditation programme. The clinics received half of the amount in advance and the rest when the clinic was accredited [10].

Attaining successful implementation of complex interventions, such as accreditation, is a challenge [11, 12]. Studies on healthcare accreditation show that implementation is influenced by a variety of factors at the individual, group, structural and organizational levels [11, 13]. In the Danish accreditation programme, only 1.2% of general practice clinics failed to become accredited [14]. Still, a qualitative study shows that the implementation varied markedly; some clinics changed only very few elements, whereas other clinics made many considerable changes in response to accreditation [10]. Moreover, some studies report that the implemented changes are not always perceived to measure up to the efforts made and that the implementation process is challenging [6, 10]. Yet, interventions with external inspections, such as accreditation, have the ability to engage and involve staff, facilitate leader engagement, improve communication and enable the creation of new networks for reflection on clinical practice. Also, inspections can contribute to creating an awareness of the inspected organisation’s current practice and performance gaps, and a commitment to change and facilitate planning and implementation of change [15]. Quantitative studies of the extent of the effect are few in a general practice setting [16, 17], and results include positive, negative and mixed results [18–21]. To our knowledge, no quantitative studies investigate associations between implementation and[16] effects of general practice accreditation.

The aim of this study is to explore how team-based implementation activities preceding accreditation are associated with self-perceived improvements in the emergency preparedness (preparedness for urgent disease and cardiac arrest) and handling of prescription renewals in a general practice setting.

## Methods

### Setting

In Denmark, general practice is the primary entry point to the healthcare system, and general practice acts as a gatekeeper to the more specialised parts of the healthcare system [22]. GPs are self-employed, and their clinics can be organised as individual clinics with only one GP or in different types of partnership practices [23]. Most clinics employ staff members, such as practice nurses and receptionists, and approximately half of the clinics have GP trainees [23].

### Study design and participants

**Fig. 1:**
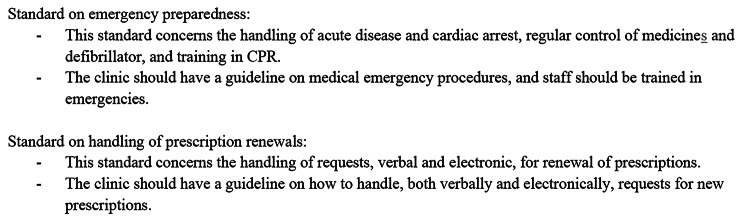
The standards of emergency preparedness and handling of prescription renewals

This is a cross-sectional study of the implementation of two specific standards in the Danish accreditation programme for general practice: emergency preparedness and handling of prescription renewals (Fig. 1). These specific standards were chosen as they are both concerned with patient care and therefore expected to be prioritized by the respondents. Though both standards are patient-centered, they are vastly different; the standard on emergency preparedness refers to rare incidents that the clinic must be prepared for, while the standard on handling of prescription renewals is a daily, routine task.

The paper conforms to the STrengthening the Reporting of OBservational studies in Epidemiology (STROBE) Standards [24].

Participants included general practice clinics conducting their accreditation survey between 27 and 2016 and 31 December 2018. After the accreditation survey, the participants received a questionnaire about their experiences with the accreditation process. The questionnaire was handed out by the visiting GP surveyor immediately after completion of the survey. The surveyor invited the GP who was most deeply involved in the accreditation to answer the questionnaire on behalf of the clinic. If the questionnaire had not been returned within two weeks, the clinics received a reminder and a new questionnaire as well as a postage-paid return envelope by regular mail.

The questionnaire consisted of 26 questions covering different aspects of the accreditation programme, including the conducted team-based implementation activities, the experience with the change process and the perceived effect of specific clinical standards included in the accreditation programme.

The questionnaire underwent a pilot test among 14 participants, including surveyors, GPs who had just completed their accreditation survey, and experienced researchers in general practice. Six of the informants gave verbal feedback to the interviewer, using cognitive interviewing techniques, during the completion of the questionnaire, whereas eight of them gave verbal feedback after completing the questionnaire [25]. In the pilot phase, the questionnaire was continually revised.

### Theoretical framework

Normalization Process Theory (NPT) provides a useful framework to gain insight into the mechanisms governing the implementation of changes in small organizations, such as general practice clinics. It was developed to describe how new interventions are introduced in various settings, and how they become routines in everyday practice [26, 27]. NPT mainly focuses on how people work together in the implementation process and is less concerned with individual intentions or attitudes [28]. The theory describes four mechanisms, which are driven by different types of team-based implementation activities that motivate and shape implementation processes [28]. [22][29]. *Coherence* describes the ways participants make sense of the intervention. *Cognitive participation* focuses on the ways participants engage in the initial implementation activities. *Collective action* relates to the way participants support the initial implementation by investing skills and resources in the intervention. Finally, *reflexive monitoring* refers to the individual and collective assessment of the implementation and how it affects the participants [29]. Each of the four constructs contain multiple sub-constructs exploring different aspects of the main construct.

The Normalisation MeAsure Development (NoMAD) instrument derives from NPT and can be used to quantitatively measure team-based implementation activities and their outcomes [28, 30]. It consists of 25 questions covering all NPT sub-constructs which assess the team-based implementation activities from the perspective of the professionals who are directly involved in the activities [28, 30].

### Team-based implementation activities

In this study, we explored three specific NPT sub-constructs within implementation: Communal specification (coherence), interactional workability (collective action), and initiation (cognitive participation). To operationalize the sub-constructs, three NoMAD questions were included in the questionnaire. A professional interpreter translated the questions and, together with the researchers, made a culturally adaptation to fit a GP setting and the context of the study (Table 1).

**Table 1:** NPT constructs and sub-constructs used to explore team-based implementation activities

NPT construct	NPT sub-construct	Team-based implementation activity (phrasing in the questionnaire)
Coherence	Communal specification: Whether people can build a collective understanding of the purpose of the intervention.	We have a common understanding of the purpose of preparing the guidelines.
Collective action	Interactional workability: Whether people can work with the intervention to perform the tasks required in their role.	The preparation of the guidelines could easily be integrated into our way of working.
Cognitive participation	Initiation: Whether key individuals are working to drive the intervention forward	Did you assign a key person for the work with the standards?

Coherence and collective action were assessed on a five-point Likert scale comprising ‘mainly agree’, ‘partly agree’, ‘neither agree nor disagree’, ‘partly disagree’ or ‘mainly disagree’. In the analyses, they were dichotomised and ‘occurrence of implementation activity’ included the first two response options. Cognitive participation was assessed through a Yes/No question, with the option to report the professional background of potential key person(s).

### Self-perceived improvements

To investigate whether the emergency preparedness had improved after the accreditation process, the respondents were asked, “How is the emergency preparedness in your clinic today compared to the time before the work with the standard?” Similarly, to evaluate if the handling of prescription renewals had improved after the accreditation process, the respondents were asked, “How is the handling of prescription renewal in your clinic today compared to the time before the work with the standard?” For both questions, the respondents could select ‘better’, ‘just as good’ or ‘worse’. In the analysis, ‘better’ indicated improvements, whereas ‘worse’ and ‘just as good’ indicated no improvements.

### Practice characteristics

Practice characteristics such as practice type, number of GP partners, number of staff, training site for junior GPs and administrative region were included as potential predictors, as they were considered to be potentially influencing the team-based implementation activities [7, 31]. Since GPs were asked to answer the questionnaire on behalf of the clinic, no individual data was collected. Practice type was categorised as single-handed or group practice, where group practice was defined as clinics with more than one GP partner and/or formalised cooperation with other clinics. Number of GP partners, and number of staff were included as continuous variables. Training site for junior GPs was categorised as a dichotomous variable indicating whether the clinic had GP trainees (Yes/No). Geographical location was based on the five Danish administrative regions: North Denmark Region, Capital Region of Denmark, Region Zealand, Region of Southern Denmark and Central Denmark Region.

### Statistical analysis

Descriptive statistics were performed to characterize the dataset and assess the frequency of implementation activities and self-perceived improvements.

Logistic regression analyses tested associations between team-based implementation activities and perceived improvements in the emergency preparedness and handling of prescription renewals. The hypothesis is that clinics who engage in team-based implementation activities preceding accreditation are more likely to experience improvements in their emergency preparedness as well their handling of prescription renewals.

Associations were calculated as unadjusted as well as adjusted odds ratios (ORs). The adjusted statistical model included the crude improvement rate adjusting for implementation activities, practice type, number of GP partners, number of staff, GP trainees in clinic and administrative region. All variables in the adjusted model were entered in one step. The analyses were restricted to respondents with complete data on all variables in the models. Spearman’s correlation coefficients were used to test for multicollinearity. No strong collinearity was found between independent variables.

The 95% confidence intervals (CIs) were calculated, and P values of ≤ 5% were considered statistically significant. Data were analysed using Stata (version 16.1).

## Results

Between 27 and 2016 and 31 December 2018, 1,230 clinics conducted their accreditation survey. A total of 920 clinics returned the questionnaire (74%). Of these, 721 clinics filled out the questionnaire which was handed out at the survey, and 199 clinics answered the reminder questionnaire. Table 2 shows the characteristics of the responding clinics.

**Table 2:** Characteristics of the respondents (total n = 920)

Characteristics	N	%
Practice type	909	
Single-handed practice	281	31
Group practice	628	68
Missing	11	1
Number of GP partners	903	
1	372	40
2	237	26
3	165	18
>3	129	14
Missing	17	2
Training site for junior GPs	907	
Yes	596	65
No	311	34
Missing	13	1
Number of staff members in clinic	920	
0–2	367	40
3–4	280	30
>4	273	30
Missing	0	0
Administrative region	920	
North Denmark Region	81	9
Central Denmark Region	212	23
Region of Southern Denmark	198	22
Region Zealand	130	14
Capital Region of Denmark	299	33
Missing	0	0
Questionnaire was filled in	917	
Alone	672	73
With colleagues	245	27
Missing	3	0.3

### Self-perceived improvements

In almost half of the clinics (48%), the respondents perceived to have obtained a better emergency preparedness compared to before. In the remaining clinics, no changes were perceived, except for one clinic in which the respondents reported to do worse (Table 3). In around a third (31%) of the clinics, the respondents reported that prescription renewals were handled better after compared to before the accreditation process. In only one clinic, the handling of prescription renewals was reported to be worse while the remaining respondents reported to experience no difference (Table 3).

**Table 3:** Perceived improvements in the emergency preparedness and handling of prescription renewals

	Better n (%)	Just as good n (%)	Worse n (%)	n*
Emergency preparedness	440 (48)	474 (52)	1 (0.1)	915
Handling of prescription renewals	280 (31)	634 (69)	1 (0.1)	915

*Five respondents did not answer the questions

### Team-based implementation activities

**Fig. 2:**
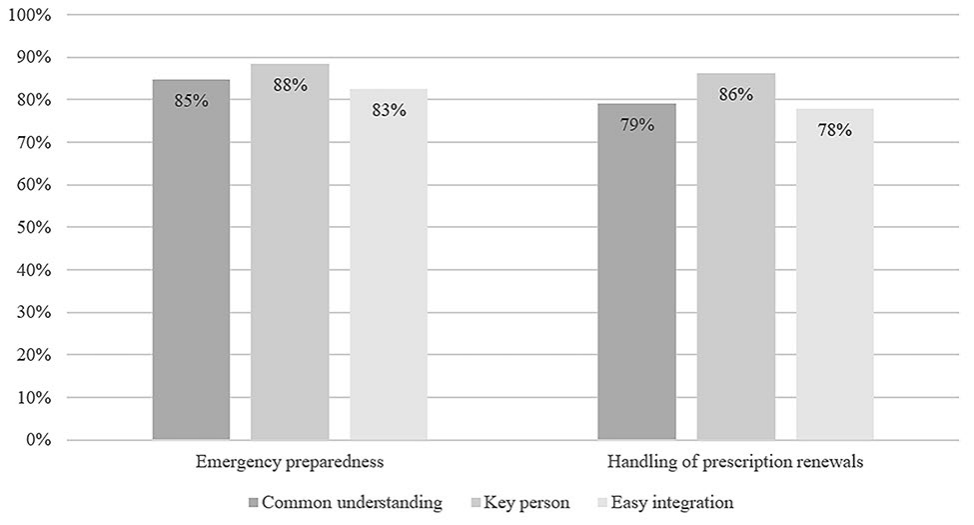
Frequency of clinics reporting team-based implementation activities

Obtaining a common understanding of the purpose of preparing the guidelines was frequently reported (85% and 79%). The most commonly reported team-based implementation activity was the assignment of a key person responsible for working with the standards (88% and 86%) (Fig. 2). Finding it easy to integrate changes into existing working procedures was reported by 83% and 78% of the clinics (Fig. 2).

Most often, the role as key person was held by one or more GPs (56% and 63%). In some clinics, the role was shared by GPs and staff (30% and 63%), whereas the key person was one or more of the staff members in few clinics (6% and 7%).

### Associations between implementation activities and improvements

The univariate logistic regression analysis showed that all team-based implementation activities were significantly associated with perceived improvements, except for the association between assigning a key person and improvements in prescription renewals (Table 4). The associations were still significant after adjusting for practice characteristics.

**Table 4:** Association between team-based implementation activities and perceived improvements

	Emergency preparedness OR (95% CI)^a^		Prescription renewals OR (95% CI)^a^	
	Crude	Adjusted^b^	Crude	Adjusted^b^
n/missing	899/21	872/48	889/31	865/55
Common understanding	4.41* (2.71–7.19)	5.07* (3.06–8.40)	4.03* (2.34–6.96)	3.66* (2.07–6.46)
Key person	1.75* (1.10–2.78)	1.95* (1.19–3.19)	1.38 (0.84–2.27)	1.24 (0.74–2.08)
Easy integration	1.76* (1.17–2.63)	1.88* (1.24–2.85)	2.27* (1.42–3.63)	2.34* (1.44–3.79)

^a^OR (95% CI): Odds ratio, 95% confidence interval

*p < 0.05

^b^Adjusted by practice type, number of GP partners, number of staff, GP trainees in clinic, and administrative region

Obtaining a common understanding of the purpose of implementing changes according to the accreditation standard was found to have the strongest association with perceived improvements (OR = 5.07 (3.06–8.40) regarding the emergency preparedness and OR = 3.66 (2.07–6.46) regarding handling of prescription renewals). Easy integration of changes was also significantly associated with improvements in both emergency preparedness (OR = 1.88 (1.24–2.85)) and handling of prescription renewals (OR = 2.34 (1.44–3.79)). Assigning a key person responsible for working with the standard was only significantly associated with improved emergency preparedness (OR = 1.95 (1.19–3.19)) (Table 4). Additional file 1 shows the full adjusted model with all covariates.

## Discussion

### Main findings

Around 80% of the clinics had performed several specific, team-based implementation activities to fulfil the criteria for accreditation. Almost half of the clinics (48%) reported perceived improvements in the emergency preparedness, and 30% reported perceived improvements in the handling of prescription renewals. Very few respondents experienced impairment of their work procedures.

For both emergency preparedness and handling of prescription renewals, around 80% of the clinics reported to have obtained a common understanding of the purpose of preparing the guidelines, and that the preparation of the guidelines could be easily integrated into their way of working. The team-based implementation activities also included assignment of a key person which was reported by 86-88%. This role was seldomly delegated exclusively to a staff member.

The results revealed significant associations between the three types of team-based implementation activities and perceived improvements in the emergency preparedness and handling of prescription renewals. Obtaining a common understanding of the purpose of implementing changes according to the accreditation standard was found to have the strongest, significant association with perceived improvements. Finding it easy to integrate changes into existing working procedures was also significantly associated with improvements, whereas assigning a key person responsible for working with the standard was only significantly associated with improved emergency preparedness.

### Strengths and limitations

An important strength of this study includes the high response rate compared to other questionnaire studies in this setting [32, 33]. Another strength includes hand distribution of the questionnaire by GP surveyors to all general practices in the study period immediately after their survey visit. In general, GP surveyors in Denmark succeeded at establishing a trust-based relation to GPs and GP staff [34] and are therefore valid distributers of the questionnaire. An important limitation of the study is the study design since cross-sectional studies do not allow us to draw firm causal conclusions. Another limitation is the embedded risk of recall bias as the experiences with both the team-based implementation activities and the perceived improvements were measured some time after the actual implementation processes. To address the risk of recall bias, we asked the GP most involved in the accreditation process to complete the questionnaire.

In the interpretation of the findings, it should also be borne in mind that the study explored the first round of accreditation in Danish general practice. The implementation activities and the perceived effects might have been different if the clinics were familiar with the concept of accreditation [18].

Furthermore, only two of the 16 accreditation standards were explored in this study. Both standards are about patient care and were chosen based on perceived clinical relevance. The findings might reflect this, and the effects of team-based implementation could be different for accreditation standards focusing on organization and management. Though most results showed associations between team-based implementation activities and self-perceived improvements, assigning a key person responsible for working with the standard was only significantly associated with improved emergency preparedness and not with prescription renewals. A possible explanation might relate to the fact that emergency preparedness refers to rare incidents, whereas prescription renewals is a daily, routine task where GPs and staff already must be expected to have more well-defined roles. Thus, a study from general practice found that implementation processes might be different for daily routine work compared to infrequent processes [35]. Assigning a key person to take care of prescription renewals might therefore be less important.

Accreditation is a highly complex process, which takes place in a complex clinical reality. Although accreditation has been studied in multiple settings [1, 18, 36], a link between accreditation and improvements in the quality of care has not been fully established [21, 37]. Also, it is difficult to ensure that measured effects can be attributed to accreditation. In this study, we sought to increase the content validity of the questions regarding perceived effects by asking questions on two specific accreditation standards.

The NoMAD instrument was chosen as a means for measuring implementation activities. In the current study, only three NoMAD questions were included as proxy measures for team-based implementation of accreditation in general practice; therefore, they should be seen as a simplification of the highly complex concept of implementation activities. The relevance of the NoMAD instrument is supported by its use in other recent studies, also in general practice [38, 39]. Furthermore, the instrument has been translated to a range of other languages, including a Danish version launched after the initiation of our data collection. Only minor differences exist between the translated version of the questions in our questionnaire, which was used in this study, and the official Danish version of the NoMAD instrument [40].

### Comparison with literature

Studies of Danish GPs’ experiences with accreditation have found large variations in the use of external implementation support during the accreditation process [7], as well as their in-clinic implementation activities and the impact of accreditation [10]. In our study, the large majority of the clinics had been engaged in specific, team-based implementation activities. However, it is not possible to assess the thoroughness of these activities.

In our study, we found that improvements in work procedures were significantly more likely to appear if any of the specific, team-based implementation activities were conducted. This is in line with other studies, finding that a shared understanding among staff [35, 41] and assigning a key person for the implementation activities [41, 42] were important factors for successful implementation of accreditation standards or other quality interventions in general practice. However, other factors may also influence the implementation activities and the effect of the accreditation programme, e.g. that the Danish programme is mandatory [13].

Implementation and effectiveness can be influenced by the pre-existing motivation to comply with a top-down mandatory intervention [43] such as an accreditation programme, which can be criticized for causing a focus on bureaucratic exercises rather than improving healthcare [44]. Thus, a favourable perception of accreditation has been identified as an enabling factor for implementation [13]. A previous study found widespread scepticism prior to the implementation of accreditation standards in general practice in Denmark [9]. This individual scepticism might influence implementation activities as well as perceived effects of accreditation. Perhaps scepticism is mostly related to the accreditation standards that are perceived as bureaucratic (e.g. standards concerning management) and less related to the more directly clinically relevant standards which are included in our study.

### Implications

We found that the percentage of clinics engaging in specific, team-based implementation activities was similar for both emergency preparedness and handling of prescription renewals. Moreover, the association between team-based implementation activities and perceived effects was to a large degree similar for both procedures. This suggests that our findings might also apply to the implementation of other accreditation standards dealing with clinical behaviour, and most likely also in other quality development initiatives aiming at improving clinical behaviour in general practice. Therefore, facilitating team-based implementation activities such as those explored can increase the likelihood of improvements in working procedures and quality of care.

## Conclusion

Clinical quality initiatives that involve collaboration within a practice team are more likely to cause improvements if specific team-based implementation activities are conducted. It is particularly important to facilitate a common understanding of the purpose of the initiative, but also assigning a key person responsible for the quality work and finding it easy to integrate changes into existing working procedures is important. Therefore, external support for quality initiatives aiming at the practice level in general practice should facilitate such team-based activities.

## Electronic supplementary material

Below is the link to the electronic supplementary material.

Supplementary Material 1
File name: Additional file 1.
File format: Microsoft Word Document (.docx).
Title of data: Association between team-based implementation activities and perceived improvements (full model with all predictors).
Description of data: Adjusted model including all predictors in the logistic regression analyses.

## Data Availability

The datasets generated and/or analysed during the current study are not publicly available due to the lack of consent of participants but are available from the corresponding author on reasonable request.

## Abbreviations

GP: General practitioner
IKAS: (Danish) Institute for Quality and Accreditation in Healthcare
NoMAD: Normalisation MeAsure Development
NPT: Normalisation Process Theory
