# Supplementary material for: What GPs do to meet accreditation standards – implementation activities and perceived improvements attributed to general practice accreditation
Source: BMC Prim Care. 2022 Oct 15;23:265. doi: 10.1186/s12875-022-01864-y (PMC9571477; doi:10.1186/s12875-022-01864-y)
Supplement: Supplementary file 1 — Supplementary Material 1 File name: Additional file 1. File format: Microsoft Word Document (.docx). Title of data: Association between team-based implementation activities and perceived improvements (full model with all predictors). Description of data: Adjusted model including all predictors in the logistic regression analyses. [file 12875_2022_1864_MOESM1_ESM.docx]

**Additional File 1**

| **Association between team-based implementation activities and perceived improvements (full model with all predictors)** | | | | |
| --- | --- | --- | --- | --- |
|  | Emergency preparedness | | Prescription renewal | |
|  | Crude OR  (95% CI) | Adjusted OR  (95% CI) | Crude OR  (95% CI) | Adjusted OR  (95% CI) |
| n/missing | 899/21 | 872/48 | 889/31 | 865/55 |
| Common understanding | 4.41* (2.71-7.19) | 5.07* (3.06-8.4) | 4.03* (2.34-6.96) | 3.66* (2.07-6.46) |
| Key person | 1.75* (1.10-2.78) | 1.95* (1.19-3.19) | 1.38 (0.84-2.27) | 1.24 (0.74-2.08) |
| Easy integration | 1.76* (1.17-2.63) | 1.88* (1.24-2.85) | 2.27* (1.42-3.63) | 2.34* (1.44-3.79) |
| Practice type: |  |  |  |  |
| Single-handed practice |  | 1 |  | 1 |
| Group practice |  | 1.42 (0.96-2.09) |  | 1.74* (1.12-2.7) |
| Number of GP-partners^b^ |  | 0.82 (0.71-0.95) |  | 1.04 (0.90-1.21) |
| Number staff members in clinic^b^ |  | 1.00 (0.96-1.05) |  | 1.04 (0.98-1.06) |
| Training site for junior GPs: |  |  |  |  |
| No |  | 1 |  | 1 |
| Yes |  | 0.76 (0.54-1.07) |  | 0.99 (0.68-1.44) |
| Administrative region: |  |  |  |  |
| North Denmark Region |  | 1 |  | 1 |
| Capital Region of Denmark |  | 1.92* (1.08-3.38) |  | 1.13 (0.61-2.11) |
| Region Zealand |  | 1.53 (0.83-2.87) |  | 1.09 (0.55-2.16) |
| Region of Southern Denmark |  | 1.1 (0.61-1.98) |  | 1.13 (0.6-2.15) |
| Central Denmark Region |  | 1.19 (0.67-2.13) |  | 1.03 (0.55-1.94) |

OR: Odds ratio; 95% CI: 95% confidence interval
*p <0.05

^b^The unit in these analyses is per partner/staff member
